# Hospitals and Lifeboats

**Published:** 1891-04-11

**Authors:** 


### Passing Topics.

### HOSPITALS AND LIFEBOATS.

It is our contemporary the *Globe* which, with characteristic neatness and precision, puts the case for the lifeboats, and reminds its readers of certain salient features possessed in common by our hospitals and the Royal National Lifeboat Institution. The *Globe* points out first the work performed by the Institution last year, with its splendid record of 773 lives saved upon our coasts—555 by its direct, and 218 by its indirect instrumentality—and considers that the country has every reason to be well satisfied with the manner in which "this great private organization accomplishes a task that in other lands would be undertaken, if undertaken at all, by the State." Then comes an examination of the financial position, and a revelation of the inevitable deficit.

The income of the Institution in 1890 amounted, we are told, to £42,523, a very respectable total; but the expenditure during the same period, including certain exceptional items, was no less than £75,877. While persuaded of the necessity, obvious enough we fear, to find means to increase the income, our contemporary does not favour the idea of a permanent State convention, but asks why there should not be a Saturday or Sunday lifeboat collection every year, adding that "the case runs on all fours with that of the hospitals; in both instances a grand work of humanity is performed by self-managed institutions."

It would be satisfactory to know that this was the conclusion of the whole matter, but, unhappily, the parallel holds good a little farther. The grand work of humanity—and who can question its grandeur?—is in both cases incomplete and inadequate. It is not apportioned to the work to be done, but only to the means available. If the Lifeboat Institution could reckon upon an income of, say, £100,000, it could double its boats, and, inferentially, it could save double the number of lives. Similarly, if the hospitals were not hampered by want of means, they could offer more than the 5,000 beds which, we are told, are available for the non-pauper sick poor of a population of five millions.

We believe, too, that a large proportion of the boats of the Association if not non-efficient are at least of more or less antiquated build, and that were there no difficulties about ways and means, a whole fleet of steam propelled boats would be immediately brought into the service. In like manner, if the hospitals possessed any funds for the purpose, several old, unsuitable, and presumably unsanitary buildings would be replaced by others fully equipped with every modern requirement.

We may parry the question as we will by time-honoured sentiment and praiseworthy preference for action essentially philanthropic, but the inquiry will obtrude itself, how far are we justified in sacrificing the health or the life of a single individual for the mere satisfaction of sustaining a record of voluntary work unleavened by an iota of "State" help? The National Lifeboat Institution is a splendid illustration of the power of unaided benevolence. Its history is a record of noble efforts and as noble achievements. But if it can be demonstrated that one life has been lost because the Institution's means are inadequate to girdle all our coasts, the system which permits the work to be carried on unaided is obviously condemned. Voluntary effort must be equal to the whole work to be done or it must consent to be supplemented or to give place to something less magnificent and less amiable but more complete and more effective. The decision rests with the public. Are they prepared to pay for the sentiment they magnify? Like many other beautiful things, it costs something to maintain.

